# Prevalence of Preterm Birth and Perinatal Outcomes in a Tertiary Hospital in Malaysia

**DOI:** 10.7759/cureus.55284

**Published:** 2024-02-29

**Authors:** Zahirrah Begam Mohamed Rasheed, Jesrine Hong, Hannuun Yaacob, Siti Zawiah Omar

**Affiliations:** 1 Department of Craniofacial Diagnostics and Bioscience, Faculty of Dentistry, Universiti Kebangsaan Malaysia, Kuala Lumpur, MYS; 2 Department of Obstetrics and Gynaecology, Faculty of Medicine, Universiti Malaya, Kuala Lumpur, MYS; 3 Department of Decision Science, Faculty of Business and Economics, Universiti Malaya, Kuala Lumpur, MYS

**Keywords:** risk factors, neonatal mortality and morbidity, perinatal outcome, prevalence, preterm birth

## Abstract

Background

Preterm birth (PTB) is defined as neonates that are born alive >22 weeks of gestation and <37 weeks of gestation. Because of the immaturity of different organ systems, 14.84 million newborns worldwide are born prematurely, which is the largest contributing factor to mortality and morbidity. Although studies have been conducted in this field, the magnitude of PTB is a major issue in most developing countries including Malaysia.

Objective

To assess the prevalence of PTB and the perinatal outcome among women delivered in a tertiary university hospital in Malaysia.

Methods

This was a cross-sectional study evaluating all singleton live births weighing>500g and delivered at >22^+1^ weeks of gestation between January 2015 and December 2019 in Universiti Malaya Medical Centre (UMMC), Kuala Lumpur, Malaysia. Data were collected from the hospital's recorded birth registry. The primary outcome was the PTB rate. Data were entered and analysed using Statistical Product and Service Solutions (SPSS) (version 28.0; IBM SPSS Statistics for Windows, Armonk, NY).

Results

A total of 26,022 singleton live births were reported for the period 2015-2019. PTB rates showed a sharp 6% decrease from 2015 to 2016, after which the trend was inconsistent until 2019. The risk of preterm babies being admitted to the neonatal intensive care unit (NICU) or the ward compared to the risk of neonatal mortality increases for babies of identified sex, delivered via caesarean, and with a birth weight between 2 and 3 kgs. Babies born at a gestational age between 22^+1^ and 33^+6^ have a higher risk of neonatal mortality compared to late preterm babies.

Conclusions

The PTB incidence trend was inconsistent from 2015 to 2019 in a tertiary university hospital in Malaysia, with a far higher prevalence compared to national data. The high NICU admission and mortality rates among preterm infants mean urgent strategies and policies are needed to improve perinatal outcomes.

## Introduction

Preterm birth (PTB) is defined as the birth of a live infant before 37 completed weeks of gestation [1]. Globally, around 15 million neonates are born preterm each year, with the worldwide PTB rate ranging from 4% to 16%, although this is higher in low- and middle-income countries [2]. As the leading cause of under-five mortality, PTB has been the focus of the World Health Organization (WHO) through the Sustainable Development Goals (SDG) and Millennium Development Goals (MDG). As pregnancy complications are related to various aetiologies and underlying factors, PTB can be divided into spontaneous or medically indicated PTB [3].

In Malaysia, both the public and private sectors provide maternity care from conception to delivery. In the public sector, the primary care level provides pre-conceptional and antenatal care services, through which screening-indicated high-risk mothers are referred to district or state hospitals for further management. According to National Obstetrics Registry (NOR) data, Malaysia demonstrated a decreasing birth incidence trend from 2016 to 2017 [4] and an increase from 2018 to 2020 [5]. These incidence data were captured from various hospitals nationwide.

A study by Sutan et al. on epidemiological PTB data from 2011 to 2015 revealed an increase in the PTB rate and risk factors associated with this outcome [6]. The data from the Sutan et al. study were gathered from a single-centred tertiary hospital that performs 15-20% of the overall in-vitro fertilisation procedures in Malaysia. These intriguing data demonstrate the importance of epidemiological studies involving various centred population research to identify the risk factors contributing to PTB, as well as the development of effective prevention interventions in the Malaysian population setting. Therefore, the current study provides evidence of the prevalence of PTB and neonatal outcomes of preterm infants born at Universiti Malaya Medical Centre (UMMC). UMMC is one of the main tertiary referral centres located in Kuala Lumpur and is a government-funded teaching hospital with an annual birth rate of between 4,000 and 5,000. Epidemiological data were gathered from the labour room admission records and the neonatal intensive care unit (NICU) admission records.

## Materials and methods

Study design and setting

A hospital-based descriptive cross-sectional study was conducted using information obtained from delivery records between January 2015 and December 2019. The chosen centre was UMMC, a tertiary government-funded hospital with nearly 5,000 annual deliveries. It is located in Kuala Lumpur, a city with a total population of eight million. As a teaching hospital for Universiti Malaya (UM) and a provider of referral-level obstetrics and neonatal care services, UMCC is one of the busiest maternity centres and handles many high-risk pregnancies, including PTB.

Data collection

Data were collected once ethical approval was granted by the Medical Research Ethics Committee of UMMC (MREC ID: 20201124-9238). All mothers who had had single live births at UMMC from January 2015 until December 2019 were identified using birth registration records held at UMMC. The information collected included gestational age at delivery, ethnicity, mode of delivery, sex of the infant, birth weight, and postdelivery status of the infant. Gestational age was calculated using the date of the last menstrual period (LMP) and first-trimester dating scan [7]. Defined as a birth occurring at a gestation of less than 37 completed weeks, PTB was further characterised as extremely preterm (22+0-27+6 weeks), very preterm (28+0-31+6 weeks), moderately preterm (33+0-36+6 weeks), and late preterm (33+0-36+6 weeks) [3].

All singleton birth data were collected, although only live births above 22+0 weeks and above 500 grams were recorded. Any live births below 22+0 weeks of gestation and below 500 grams were considered miscarriages [8] and then excluded. Multiple-pregnancy data were also excluded. A total of 2,787 PTB cases were recorded, but two cases were dropped during the subsequent analysis as the mode of delivery and birth weight data were missing.

Data analysis

Data were entered into Excel sheets and cleaned before being analysed using Statistical Product and Service Solutions (SPSS) (IBM SPSS Statistics for Windows, Armonk, NY). Descriptive statistics, such as frequencies and percentages, were used to provide an overview of the variables. Additionally, visualisation in the form of bar charts will also be included to illustrate trends and distribution. Furthermore, multinomial logistic regression analysis was conducted owing to its framework, which allows for the simultaneous examination of the effects of multiple independent variables on a categorical dependent variable with three or more categories. Given the three categories within perinatal outcomes (i.e., mortality, admission to NICU, and admission to ward/others), multinomial logistic regression was particularly well-suited for understanding the relationship between perinatal outcomes and other variables. In this multinomial regression model, mortality was selected as the reference category for the dependent variable. The independent variables include gestational age at delivery, mode of delivery, sex of the infant, and birth weight. A significance level of p<0.05 was used to determine the statistical significance of the observed relationship within the regression model.

## Results

Total deliveries and prevalence of preterm births (PTBs)

**Figure 1:**
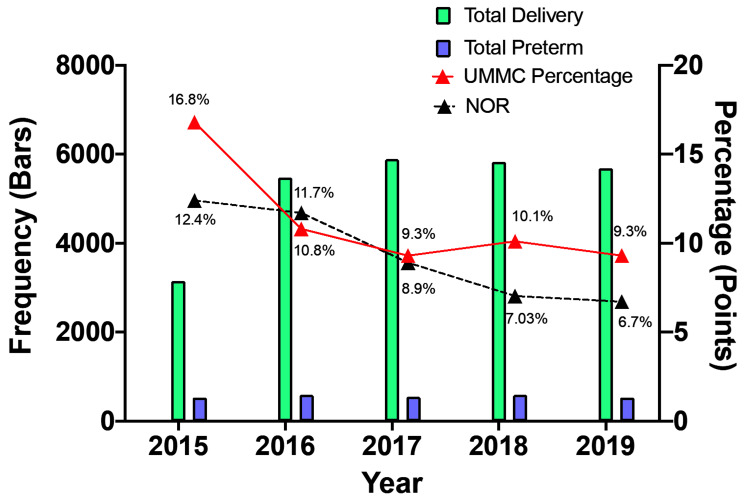
Total deliveries and singleton PTBs from 2015to 2019 at UMMC. The green bar charts represent the total deliveries in UMMC, and the blue bar charts are the total PTB in UMMC. The line charts are the PTB rates for UMMC and NOR in red and black, respectively.

The bar chart in Figure 1 illustrates the trends in total deliveries and total singleton PTB between 2015 and 2019 at UMMC. Analysis of the total deliveries at UMMC shows a fluctuating trend over the five-year period. The lowest number of deliveries was reported in 2015 (N=3,155), followed by an increase in 2016 (N=5,476) and then a further increase in 2017 (N=5,885). Subsequently, stagnant total deliveries were observed in 2018 (N=5,823), with a slight decline in 2019 (N=5,683). Despite the irregular total deliveries trend over five years, the trend in PTB from singleton pregnancies is relatively consistent, with the highest frequency reported in 2018 (N=594). Additionally, Figure 1 illustrates the trend for the ratio of total deliveries to total PTB for UMMC and NOR in red and black, respectively. In 2015, it was found that UMMC recorded the highest PTB rate at 16.9%, while NOR had a rate of 12.4%. For UMMC, subsequent years show fluctuations, with rates ranging between 9% and 10% annually from 2016 to 2019. Conversely, the PTB rate for NOR shows a declining pattern from 2016 to 2019.

Demographic characteristics of PTB

The descriptive statistics for the demographic, delivery characteristic, and neonatal outcome data collected from UMMC are presented in Table 1. Between 2015 and 2019, those of Malay ethnicity (N=1,635; 58.7%) recorded the most PTB cases at UMMC, followed by those of Chinese (N=466; 16.7%), Indian (N=357; 12.8%), and other ethnicities (N=327; 11.7%). The spontaneous vaginal delivery (SVD) rates were N=1,236; 44.4%, while caesarean delivery rates were N=1,338; 48.0%. In terms of the sex of the infant, a higher PTB rate was recorded among males (N=1,524; 54.7%) compared to females (N=1,251; 44.9%). Most neonates were born weighing between 2 kg and 3 kg (N=1,560; 56.0%), whereas 31.8% (N=886) of them were born weighing less than 2 kg. Over half of the preterm neonates were admitted for special care (N=1,754; 63.0%), while 30.3% (N=844) were admitted to the NICU and 6.7% (N=187) could not be revived after birth. A large number of the neonates were born at late preterm (N=2,056; 73.8%). However, extreme preterm (N=162; 5.8%) and very preterm (N=283; 10.2%) births collectively represented a higher rate compared to moderate PTB (N=284; 10.2%).

**Table 1:** Distribution of the demographics, delivery characteristics, and neonatal outcomes of preterm neonates. ^a^Assisted vaginal delivery includes forceps and vacuum assisted. ^b^Others include vaginal breech delivery. ^c^Others include infant transfer to other hospitals. SVD: spontaneous vaginal delivery; NICU: neonatal intensive care unit.

Variables	Category	Frequency (N)	Percentage (%)
Ethnicity	Malay	1,635	58.7
	Chinese	466	16.7
	Indian	357	12.8
	Others	327	11.7
Mode of delivery	SVD	1,236	44.4
	Caesarean delivery	1,338	48.0
	^a^Assisted vaginal delivery	142	5.1
	^b^Others	69	2.5
Sex	Male	1,524	54.7
	Female	1,251	44.9
	Ambiguous	10	0.4
Birthweight	500-2,000g	886	31.8
	2,001-3,000g	1,560	56.0
	3,001-5,500g	339	12.2
Baby’s postdelivery status	Neonatal mortality	187	6.7
	NICU	844	30.3
	^c^Ward/others	1,754	63.0
Preterm severity	Extremely preterm	162	5.8
	Very preterm	283	10.2
	Moderately preterm	284	10.2
	Late preterm	2,056	73.8

Neonatal outcomes

**Figure 2:**
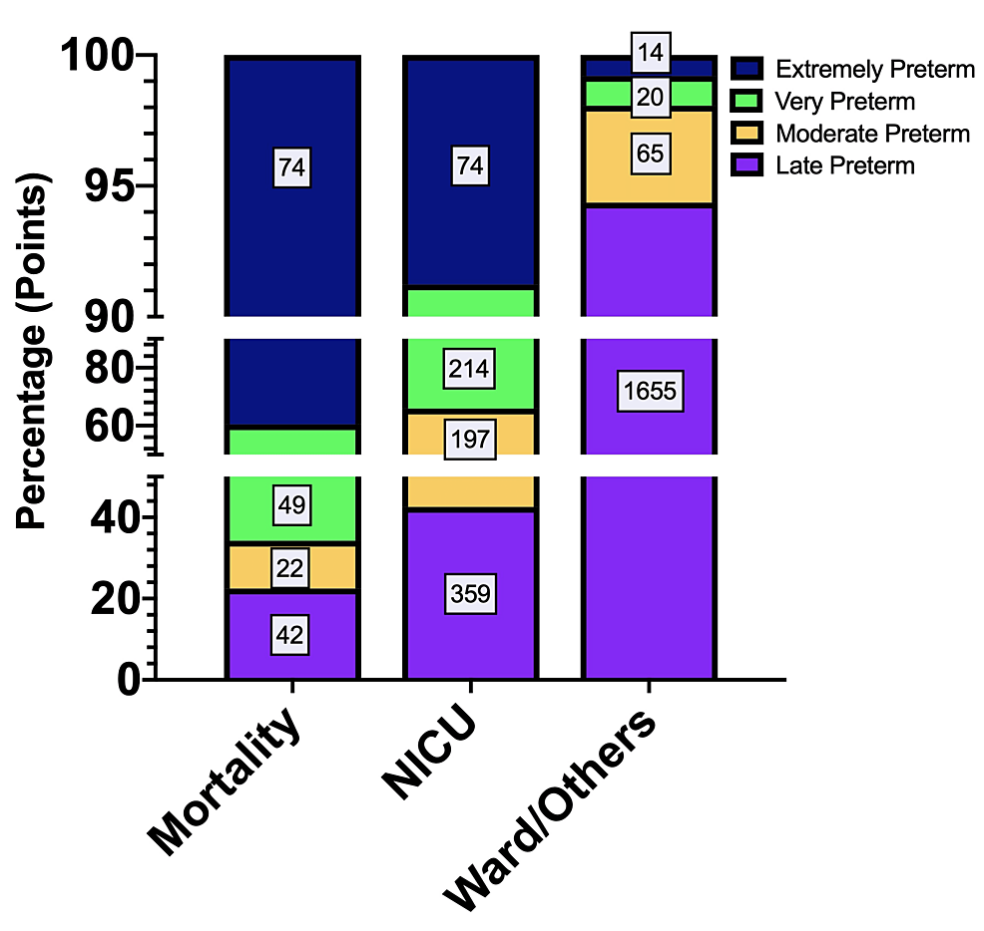
Neonatal mortality, NICU-admitted, and ward-/other-admitted preterm neonates from 2015 to 2019. The preterm neonate outcomes were categorised based on the preterm severity.

Stratifying the preterm severity categories into neonatal outcomes after delivery, 40% of neonatal mortality was observed in the extremely preterm group followed by 26.2% in the very preterm group. For the NICU category, late preterm neonates showed the highest admission rate at 42.5% compared to moderate preterm and very preterm neonates at 23% and 25%, respectively. Additionally, late preterm neonates also dominate the ward/others category with 94.4% compared to other preterm neonates (Figure 2).

Table 2 presents the results of multinominal logistic regression analysis examining the risk of neonates being admitted to the NICU and being admitted to the ward/others versus the reference category of neonatal mortality. All factors demonstrate statistical significance (p<0.001) indicating an association with prenatal outcomes. In the first part of the multinomial regression model, male infants (aOR: 11.995, CI: 2.197-65.490), caesarean deliveries (aOR: 8.943, CI: 3.583-22.318), and those with a birth weight of between 2 kg and 3 kg (aOR: 3.268, CI: 1.163-9.186) exhibited the greatest risk of being admitted to the NICU compared to facing the risk of neonatal mortality. However, contrasting results were observed for gestational age. Babies delivered between 22+1 and 27+6 weeks of gestation had a higher risk of mortality (aOR: 0.273, CI:0.158-0.469) instead of being at risk of admission to the NICU compared to late preterm neonates.

**Table 2:** Results from multinomial logistic regression analysis showing risk estimates associated with perinatal outcomes in NICU and ward/others, compared to neonatal mortality. *Reference category; LSCS: lower (uterine)-segment caesarean section.

Perinatal Outcomes	Variables		B	Exp(B)	Sig.	95% CI	
						Lower Bound	Upper Bound
NICU	Sex				<0.001		
		Boy	2.484	11.995	0.004	2.197	65.490
		Girl	2.267	9.651	0.009	1.769	52.647
		Undetermined*	-	-	-	-	-
	Gestational age				<0.001		
		Extremely preterm	-1.300	0.273	<0.001	0.158	0.469
		Very preterm	-0.171	0.843	0.519	0.501	1.417
		Moderate preterm	0.322	1.380	0.288	0.761	2.501
		Late preterm*	-	-	-	-	-
	Mode of delivery				<0.001		
		SVD	-0.054	0.948	0.905	0.394	2.278
		LSCS	2.191	8.943	<0.001	3.583	22.318
		Assisted device	0.489	1.631	0.368	0.562	4.731
		Others*	-	-	-	-	-
	Birthweight				<0.001		
		<2 kg	0.230	1.258	0.643	0.476	3.323
		2-3 kg	1.184	3.268	0.025	1.163	9.186
		4-5.5 kg*	-	-	-	-	-
Ward/Others	Sex				<0.001		
		Boy	1.237	3.444	0.267	0.387	30.621
		Girl	1.431	4.182	0.199	0.471	37.172
		Undetermined*	-	-	-	-	-
	Gestational age				<0.001		
		Extremely preterm	-3.222	0.040	<0.001	0.019	0.083
		Very preterm	-2.854	0.058	<0.001	0.029	0.113
		Moderate preterm	-1.691	0.184	<0.001	0.097	0.348
		Late preterm*	-	-	-	-	-
	Mode of delivery				<0.001		
		SVD	-0.0251	0.778	0.626	0.283	2.134
		LSCS	0.752	2.122	0.158	0.747	6.027
		Assisted device	-0.0440	0.644	0.477	0.191	2.169
		Others*	-	-	-	-	-
	Birthweight				<0.001		
		<2 kg	-2.961	0.052	<0.001	0.021	0.127
		2-3 kg	0.369	1.446	0.447	0.559	3.741
		4-5.5 kg*	-	-	-	-	-

The second part of the multinomial regression model is the risk of being admitted to wards/others versus neonatal mortality. Female babies (aOR: 4.183, CI: 0.471-37.172), caesarean deliveries (aOR: 2.122, CI: 0.747-6.027, 22.318), and those with a birth weight between 2 kg and 3 kg (aOR: 1.446, CI: 0.559-3.741) displayed the highest risk of being admitted to the ward/others as compared to neonatal mortality. Other variables were more prone to mortality than to being admitted.

## Discussion

PTB is rising in most countries, with the highest burden detected in low- and middle-income countries, especially those in sub-Saharan Africa and Southeast Asia [1]; therefore, Malaysia is included. The PTB prevalence in the centre examined in this study dropped by 6% between 2015 and 2016, with an inconsistent trend recorded from 2016 to 2019. The initial drop is consistent with the falling PTB trend reported by the National Obstetrics Registry (NOR) of Malaysia. The NOR also reported a 5.5% decrease in the number of live births between 2015 and 2016, which could explain the decrease in the PTB rate [4,9].

Of the middle-income countries, Malaysia’s PTB rate indicated that it experienced the lowest prevalence, compared to the rates of 13.6% in Indonesia [10], 24.3% in Egypt, 11.1% in Brazil [2], 16.0% in Nigeria [11], and 17.0% in South Africa [12]. Differences in the study context, method of gestational age assessment, sample size, and study population may have influenced the variations among the estimated prevalence of these countries. Additionally, factors such as inadequate infrastructure, technical equipment, and resources may also impede the provision of good-quality maternal care.

According to our data, babies of Malay ethnicity were more likely to be delivered prematurely. In contrast, the findings from NOR data revealed that those of Indian ethnicity preceded Malay and Chinese ethnicities in terms of the PTB distribution in Malaysia [13]. This evidence aligns with a study by Ruey et al., demonstrating that those of Indian ethnicity had a greater rate of higher-order pregnancies than the other races in Malaysia [14]. However, multiple pregnancies were not factored into this study, which would explain the disparity in ethnicity-related PTB. Nonetheless, there is mounting evidence to demonstrate a correlation between PTB and ethnicity in the United States [15], where the PTB risk associated with race is significantly higher among Black women compared to White women, based on a large systematic review [16]. Therefore, the diverse multiracial context of Malaysia is an area to be further explored.

The infants being male, mode of delivery, and birth weight were the most significant risk factors for perinatal outcomes. Previous studies have revealed that male infants have a greater risk of PTB and neonatal morbidity compared to females, who have been shown to have better health outcomes [6,17]. Many theories have been proposed to explain this connection, with several studies suggesting that the foetus's sex with the maternal hypothalamic-pituitary-adrenal axis affects foetal development and pregnancy outcomes [18]. Increased inflammatory reactions were also demonstrated in women carrying a male foetus [19], which could suggest the predisposing effect of a higher number of morbidity outcomes in male neonates who were granted further intensive care. Neonates being delivered by caesarean were also associated with an increased risk of NICU admission, with the same finding reported elsewhere [20]. Delivery by caesarean section is indicated when there is foetal compromise or when the maternal condition would detrimentally affect both the mother and foetus. Therefore, neonates are more likely to be admitted to the NICU for further specialised care [21].

Gestational age and birth weight are the most important factors in predicting the survival rates of premature infants [22] as they are more likely to experience complications. These complications are a significant factor in the direct cause of neonatal deaths, accounting for 35% of the world’s 3.1 million neonatal deaths per year [3]. A low gestational age means that the organs are immature and unprepared to support life in the extrauterine environment, thus increasing the risk of acute neonatal illnesses that would cause death [23], especially among extremely preterm and very preterm groups [24,25]. Compared to extremely preterm and very preterm infants, those of late preterm are more frequently admitted to the NICU, as seen in both this study and a recent review study [26]. As their birth weight and size are identical to those of term infants, late preterm infants are often overlooked because of their less mature physiology, limited response to the extrauterine environment, and higher risk of various morbidity, mortality, and adverse long-term outcomes [27]. Although Malaysia is demonstrating improved mortality rates because of advances in perinatal care [28], further efforts and research remain vital in reducing NICU admission and neonatal mortality rates among preterm infants.

Limitations

This study has several limitations. First, the results have a single-centre focus, but the authors believe that these data from a multi-ethnic population would be potentially generalisable to other low- and middle-income countries in the region. Misclassification of preterm severity was also possible because of the method of determining the gestational age [29]. The preterm cases reported at or referred to this hospital may not be similar to those of other tertiary hospitals as the UMMC is the main referral institution in Malaysia. Additionally, this study lacks an assessment of the comorbidities of the neonates to predict their survival, which could be added in future studies.

## Conclusions

The incidence of PTB in a tertiary referral hospital in Malaysia from 2015 to 2019 fell from 2015 to 2016, but the trend was then stagnant until 2019. The highest PTB was observed in those of Malay ethnicity, while the sex of the infant, mode of delivery, and birth weight were the significant risk factors for adverse perinatal outcomes. More enhanced interventions are needed to reduce neonatal mortality and NICU admissions of preterm infants.

## References

[1] Walani SR (2020). Global burden of preterm birth. Int J Gynaecol Obstet.

[2] Ohuma EO, Moller AB, Bradley E (2023). National, regional, and global estimates of preterm birth in 2020, with trends from 2010: a systematic analysis. Lancet.

[3] Blencowe H, Cousens S, Chou D (2013). Born too soon: the global epidemiology of 15 million preterm births. Reprod Health.

[4] Mendez-Figueroa H, Truong VT, Pedroza C, Khan AM, Chauhan SP (2016). Small-for-gestational-age infants among uncomplicated pregnancies at term: a secondary analysis of 9 maternal-fetal medicine units network studies. Am J Obstet Gynecol.

[5] Crovetto F, Triunfo S, Crispi F, Rodriguez-Sureda V, Dominguez C, Figueras F, Gratacos E (2017). Differential performance of first-trimester screening in predicting small-for-gestational-age neonate or fetal growth restriction. Ultrasound Obstet Gynecol.

[6] Sutan R, Mohamed NE, Mahdy ZA (2018). A 5 year trend and predictors of preterm births in single referral centre of the Greater Kuala Lumpur, Malaysia. International Journal of Pregnancy and Child Birth.

[7] Salomon LJ, Alfirevic Z, Bilardo CM (2013). ISUOG practice guidelines: performance of first-trimester fetal ultrasound scan. Ultrasound Obstet Gynecol.

[8] Dugas C, Slane VH (2019). Miscarriage. StatPearls.

[9] Madden JV, Flatley CJ, Kumar S (2018). Term small-for-gestational-age infants from low-risk women are at significantly greater risk of adverse neonatal outcomes. Am J Obstet Gynecol.

[10] Etil T, Opio B, Odur B, Lwanga C, Atuhaire L (2023). Risk factors associated with preterm birth among mothers delivered at Lira Regional Referral Hospital. BMC Pregnancy Childbirth.

[11] Zini ME, Omo-Aghoja LO (2019). Clinical and sociodemographic correlates of preterm deliveries in two tertiary hospitals in southern Nigeria. Ghana Med J.

[12] Zar HJ, Pellowski JA, Cohen S, Barnett W, Vanker A, Koen N, Stein DJ (2019). Maternal health and birth outcomes in a South African birth cohort study. PLoS One.

[13] (2023). National Obstetrics Registry (NOR) Malaysia. https://www.acrm.org.my/nor/.

[14] Soon Ruey CKL, Michael FW Hoong, Nur Amirah Zolkepali (2023). Multiple pregnancies - a risky affair for mothers and babies. Malaysia.

[15] Manuck TA (2017). Racial and ethnic differences in preterm birth: a complex, multifactorial problem. Semin Perinatol.

[16] Schaaf JM, Liem SM, Mol BW, Abu-Hanna A, Ravelli AC (2013). Ethnic and racial disparities in the risk of preterm birth: a systematic review and meta-analysis. Am J Perinatol.

[17] Peelen MJ, Kazemier BM, Ravelli AC (2016). Impact of fetal gender on the risk of preterm birth, a national cohort study. Acta Obstet Gynecol Scand.

[18] Al-Qaraghouli M, Fang YM (2017). Effect of fetal sex on maternal and obstetric outcomes. Front Pediatr.

[19] Ghidini A, Salafia CM (2005). Gender differences of placental dysfunction in severe prematurity. BJOG.

[20] Thanh BY, Lumbiganon P, Pattanittum P (2019). Mode of delivery and pregnancy outcomes in preterm birth: a secondary analysis of the WHO Global and Multi-Country Surveys. Sci Rep.

[21] Khasawneh W, Obeidat N, Yusef D, Alsulaiman JW (2020). The impact of cesarean section on neonatal outcomes at a university-based tertiary hospital in Jordan. BMC Pregnancy Childbirth.

[22] Sania A, Smith ER, Manji K (2018). Neonatal and infant mortality risk associated with preterm and small for gestational age births in tanzania: individual level pooled analysis using the intergrowth standard. J Pediatr.

[23] Gagliardi L, Cavazza A, Brunelli A (2004). Assessing mortality risk in very low birthweight infants: a comparison of CRIB, CRIB-II, and SNAPPE-II. Arch Dis Child Fetal Neonatal Ed.

[24] Schindler T, Koller-Smith L, Lui K, Bajuk B, Bolisetty S (2017). Causes of death in very preterm infants cared for in neonatal intensive care units: a population-based retrospective cohort study. BMC Pediatr.

[25] Stoll BJ, Hansen NI, Bell EF (2010). Neonatal outcomes of extremely preterm infants from the NICHD Neonatal Research Network. Pediatrics.

[26] Karnati S, Kollikonda S, Abu-Shaweesh J (2020). Late preterm infants - changing trends and continuing challenges. Int J Pediatr Adolesc Med.

[27] Sharma D, Padmavathi IV, Tabatabaii SA, Farahbakhsh N (2021). Late preterm: a new high risk group in neonatology. J Matern Fetal Neonatal Med.

[28] Boo NY, Chee SC, Neoh SH (2021). Ten-year trend of care practices, morbidities and survival of very preterm neonates in the Malaysian National Neonatal Registry: a retrospective cohort study. BMJ Paediatr Open.

[29] Balchin I, Whittaker JC, Steer PJ, Lamont RF (2004). Are reported preterm birth rates reliable? An analysis of interhospital differences in the calculation of the weeks of gestation at delivery and preterm birth rate. BJOG.

